# Women’s experiences of breech birth decision making: An integrated review

**DOI:** 10.18332/ejm/143875

**Published:** 2022-01-25

**Authors:** Sara E. Morris, Deborah Sundin, Sadie Geraghty

**Affiliations:** 1 School of Nursing and Midwifery, Edith Cowan University, Perth, Australia; 2 King Edward Memorial Hospital, Perth, Australia

**Keywords:** breech presentation, women’s autonomy, midwifery

## Abstract

**INTRODUCTION:**

Currently, caesarean section is the primary mode of birth for a breech presenting fetus, leading to a deskilling of clinicians and limitation of birth choices for women. The aim of this review is to present a synthesized summary of existing literature related to women’s experiences of breech birth mode decision-making.

**METHODS:**

A systematic search of the literature was conducted in April 2021, utilizing five databases to identify and obtain peer-reviewed articles meeting the predetermined selection criteria.

**RESULTS:**

Four major categories were synthesized from the integrated review: 1) Women who desire a vaginal birth may experience a range of negative emotions such as feelings of disempowerment, loss, uncertainty and a sense of isolation; 2) Women who experience a breech presentation at term experience significant pressures to conform to expectations of medical professionals and their families due to perceptions of risk related to breech birth; 3) Breech birth decision-making in a limiting system; and 4) Overall satisfaction with the decision to plan a vaginal breech birth.

**CONCLUSION:**

Women with a breech presenting fetus at term experience a complex range of emotions and internal and external pressures due to perceptions of risk around breech birth. Midwives were seen as helpful throughout the breech experience. The reduced caesarean section rate for breech, observed in studies exploring specialized care pathways or dedicated services, could reduce the incidence of Severe Acute Maternal Morbidity.

## INTRODUCTION

The caesarean section (CS) rate for breech presentation has been increasing since mid-20th century^1^. The Term Breech Trial (TBT) was a much-anticipated randomized control trial (RCT) expected to provide the answer to the long-held question: ‘What is the safest birth mode for a breech presenting fetus?’. Although, by the time the TBT was published in 2000^2^ the rate of CS for breech presentation had already surpassed 83%^2^. Despite criticism of the validity of the TBT findings, due to critiques related to several factors including recruitment, randomization, labor management protocols and the skill level of attending practitioners involved^1,3^, for many the TBT corroborated the belief that CS was indeed the safest mode of birth for breech presenting fetuses. Several studies since have shown a significantly lower risk of neonatal mortality and little to no difference in long-term developmental outcomes for breech born children, regardless of birth mode, depicting the findings of the TBT as a statistical outlier^1,3^. In spite of the CS rate for breech presentation ranging from 69% to 100% depending on the country of birth^4^, some women continue to express a preference and seek support for a vaginal birth^4,5^. Understanding women’s experiences of breech presentation and birth could highlight ways to improve clinical interactions and support for women who desire a birth outside of what has become standard management (i.e. CS)^4^. This article aims to integrate current knowledge surrounding women’s experiences of breech birth decision-making, obtained from a systematic search of the literature, in order to highlight potential practice improvements.

## METHODS

### Search strategy

**Figure 1:**
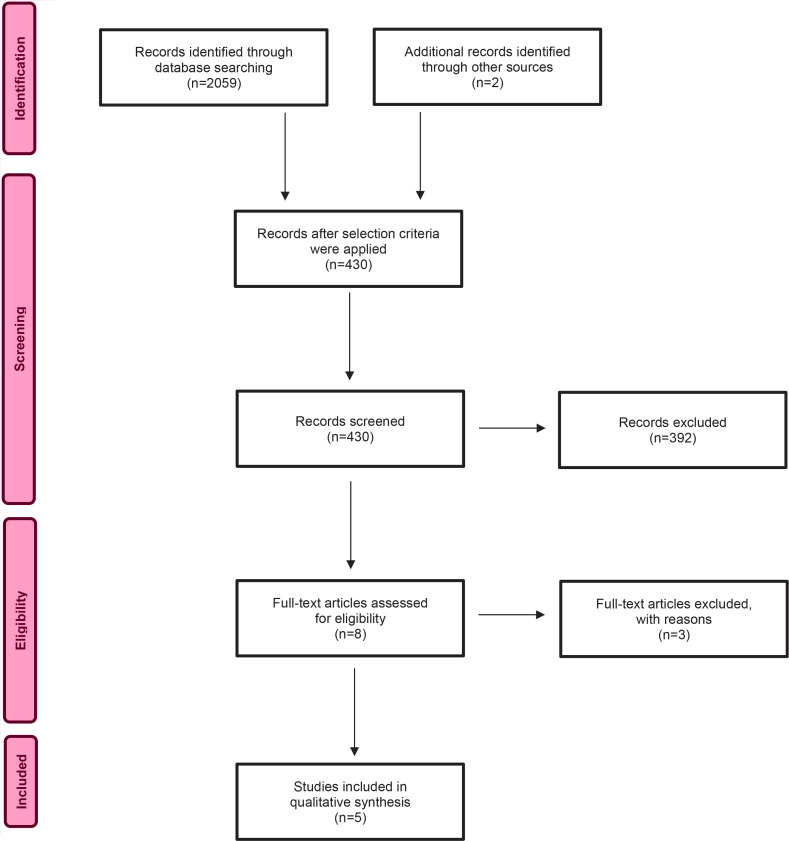
Prisma flow chart

The search objective was to identify published literature relating to the topic of interest. The following question was developed using the PICO (Population, Phenomenon of Interest and Context) mnemonic: ‘What do women (P) with a breech presentation at term report experiencing (I) in contemporary maternity care during birth mode decisionmaking (CO)?’. In order to determine the eligibility of articles for review the following criteria were established: written in English, full text, peer-reviewed articles published between 2012 and 2021 which explored women’s experiences of breech presentation. Articles were excluded if they did not meet the selection criteria, focused on only experiences or outcomes of an intervention such as External Cephalic Version (ECV) or CS. The following search terms were entered into three databases (CINHAL Plus with full text, MEDLINE, PubMed, SCOPUS) and a university library catalogue search engine (WorldSearch) in varying combinations: women, breech, birth, presentation, experience or experiences. Results were input into a PRISMA flowchart (Figure 1) to outline the search process. Each included article was reviewed and entered into a summary table (Table 1). This process aided identifying commonalities and differences between studies. The reference lists were examined for further potential studies for inclusion however; six articles were already included and 290 did not meet the selection criteria. Once completed, the details of each study were entered into the JBI SUMARI software in order to appraise, extract and synthesize the data. A total of five qualitative studies, two cross-sectional descriptive studies and one case control study were included in this review. A narrative summary is provided below.

**Table 1:** Summary of included studies

Authors and Year	Methods and Setting	Key findings
Glasø et al.^8^ 2013	Descriptive crosssectional study Norway	Participants: 299 Women, aged 24-37 years, who had planned a vaginal breech birth or requested caesarean section. Women who were selected for vaginal breech delivery (n=187) were younger, more often nulliparous and gave birth to smaller babies. Women who requested a caesarean section (n=112) became more worried when the breech presentation was diagnosed. They had a more negative initial view on breech presentation, more often took additional advice from non-professionals and trusted them more. Women who requested caesarean section reported a positive birth experience more frequently than women who were selected for vaginal delivery, whether ending as vaginal or emergency caesarean delivery. Women in both groups searched web-based information about breech delivery. We found no differences between the sources of information used.
Homer et al.^9^ 2015	Qualitative descriptive study, in-depth, semi-structured interviews (interview guide utilized) were thematically analysed NSW, Australia	Participants: 22 women, 73% primiparous, all Caucasian, most educated at a tertiary level, 41% attended a hospital that supported VBB, 55% achieved a VBB and 45% had a C/S. Four main themes with subcategories: 1) Reacting to a loss of control, loss of choice, symptoms of stress, feeling trapped, and grieving; 2) Bargaining, wanting the information given to be trustworthy, trust versus mistrust of the information, reacting to scare tactics information, the absence of good information, and needing non-emotive information; 3) Fighting the system/seeking support for a vaginal birth, courage and resolve to fight, taking control, non-fearful clinicians, and support to get back on the path to normal birth; and 4) The importance of having a go, labor as a rite of passage, labor as a rite of choice, the way women want to experience birth, for herself, for the baby.
Morris et al.^4^ 2021	Semi-structured interviews, transcribed. Free form ‘circling and parking’ style of analysis utilizing Foucauldian concepts of power and knowledge to describe observed power relations Australia	Participants: 20 women, aged 23-41 years, 85% were diagnosed antenatally with a breech presentation, 50% had a fetus in frank breech position, 25% in complete position, with the remaining either in a footling or unknown position. Women experienced 5 distinct stages throughout their experience similar to the Kubler-Ross model of grief. They did not experience these stages in any particular order and sometimes cycled through different stages multiple times. Women experienced varying degrees of disciplinary power throughout their experience. Knowledge was used as a means of enforcing disciplinary power by some clinicians and by women to ‘arm’ themselves and ‘fight’ to regain what they perceived as a loss of power and autonomy. Midwives were seen as navigators of a restrictive, medicalized healthcare system.
Petrovska et al.^10^ 2016	Quantitative results of a multinational electronic survey Multinational	Participants: 204 women from mostly European settled countries, over 40% were aged 31-35 years, over 75% had a tertiary level of education, and 63.4% experienced a vaginal birth. In total, 204 unique responses to the survey were obtained from women who had sought the option of a vaginal breech birth in a previous pregnancy. Most women (80.8%) stated that they were happy with the birth choices they made, and a significant proportion (89.4%) would attempt a vaginal breech birth in subsequent pregnancies. Less than half of women were formally referred to a clinician skilled in vaginal breech birth when their baby was diagnosed breech (41.8%), while the remainder sourced a clinician themselves. Half of the women felt supported by their care provider (56.7%) and less than half (42.3%) felt supported by family and friends.
Petrovska et al.^11^ 2017	Qualitative results of a multinational electronic survey Multinational	Participants: 204 women, the same sample as above. Qualitative results of previous study. Eight main themes: 1) Seeking the chance for a vaginal breech birth for women who attempted a vaginal birth, even if they did achieve it expressed satisfaction in knowing they tried. For women who were not given the opportunity, the sense of loss was significant. They felt disempowered despite being excited and moved by meeting their newborn; 2) Encountering coercion and fear, women reported scare tactics and judgmental attitudes from care providers and identified this as the source of their stress; 3) Putting the baby before the birth, pressure and judgement from families and friends featured strongly through the decision-making process. Accusations of putting the birth before the baby were common. Support was found on social media; 4) Overcoming obstacles in the system, women found themselves negotiating a system in conflict over vaginal breech birth. They expressed a concern over the lack of system-wide support for vagina breech birth; 5) Minimizing the opportunity for supportive clinicians to observe vaginal breech birth thus limiting skill development opportunities.; 6) Dealing with emotional wounds, stress and anxiety. For some, the day of their baby’s birth was a mostly negative experience; 7) Searching for information and support, without access to a supportive clinician women sorted information to assist them in informed decision making. The internet was used as a tool to gather and share information and build confidence in their decision-making process; and 8) Travelling across boundaries, women changed care providers and often travelled several hours to find and gain support for a vaginal breech birth.
Petrovska et al.^12^ 2017	Qualitative descriptive study NSW, Australia	Participants: 22 women, 12 had a vaginal birth and 10 had a caesarean section - same sample as in Homer et al.9. Women reported having confidence in their body to birth their baby without medical intervention which was not always shared by others. Many women reported that their families and friends accepted the dominant social discourse of vaginal breech birth being dangerous. Women reporting being told horror stories, were accused of being selfish, or mad, and putting the birth before the baby. Women report their intimate social network questioning their competency to make the decision to birth their baby vaginally. Women reported having numerous discussions with family and friends that vaginal breech birth was a reasonable alternative. Women reported that their social network often viewed caesarean section as a no-risk birth and struggled with the women’s decision against this intervention. They tried to address misconceptions about vaginal breech birth. They reported seeking further information on social media, internet searchers, etc. Seeking support and developing new social networks. They reported keeping secrets and managing their family’s anxiety.
Thompson et al.^13^ 2019	Grounded theory interviewing parents who had experienced breech presentation at term England	Participants: 12 parents (2 antenatal, 7 postnatal women, and 3 postnatal fathers, only 2 couples in the sample). Telephone interviews recorded and transcribed, analysis took place in NVivo for Mac version 11.4.0 with line-by-line coding. Two core themes: 1) Framework of influences on parents’ term breech mode of birth decision-making, internal and external influences, partner relationships, family and friend, healthcare professionals, shared experiences, time available for decision-making, personality, and personal birth culture; and 2) Mortality salience, fear of death or injury, lens through which potential influences/experiences were focused into birth mode decision-making.
Toivonen et al.^14^ 2014	Case control study Finland	Participants: 97 breech births compared to 73 cephalic births. Cases matched by age, history, mode of birth and labor/birth interventions. Responses to Childbirth Experience Questionnaire (CEQ) by women attempting a vaginal birth compared between groups. No difference in terms of birth experiences (breech vs cephalic) except in terms of maternal birth position. Women who had a breech baby reported a more positive experience with the exception of choice of analgesia, though this was not statistically significant.

### Quality appraisal

Included articles underwent quality appraisal using JBI Quality appraisal checklists for qualitative research, cross-sectional and case control studies. These checklists are available from https://jbi.global/critical-appraisal-tools.

### Data extraction and synthesis

Munn et al.^6^ describe using meta-aggregation with the purpose of meta-synthesis to gather findings from qualitative research. This is achieved by grouping findings with similar meanings into categories and amalgamating them to generate statements that adequately represent the resultant synthesis and major categories. This process of extraction and synthesis of data was used to guide this review and was completed using the JBI SUMARI software. The findings from each text were extracted and those which bore similarities were arranged into subcategories and dimensions. These were then amalgamated to form the major synthesized categories, which were agreed upon and used to synthesize information that represents what is known about women’s experiences of breech birth mode decision-making.

## RESULTS

A total of 2059 potential records were identified with 430 remaining after inclusion parameters were applied. After title and abstract review, eight were deemed suitable for full text evaluation once duplicates and exclusions were removed (Figure 1). Exclusions included opinion papers, clinicians’ experiences and an exploration of women’s experiences of decision-making for CS rather than breech presentation^7^.

### Narrative summary of included studies

Glasø et al.^8^ conducted a cross-sectional descriptive study set in Norway exploring women’s experiences of birth mode decision-making to determine potential influences. Women were considered eligible for inclusion if they had a live, singleton, term fetus in breech presentation born in one hospital between 2006 and 2010. Case exclusions included fetal malformations, successful ECV, prematurity, and multiple pregnancies. A total of 635 women were identified via the hospital register, 299 met criteria for a vaginal breech birth (VBB). Of these, 187 women planned a vaginal birth and 112 women had requested a CS. The questionnaire was sent to 293 women (six having emigrated) regarding their birth mode choice, their feelings about breech presentation and satisfaction they felt related to the support and information they received. The women’s medical records were also examined to gather demographic information and birth outcome data. The study found women who planned a VBB were younger (mean age 29 years), more often nulliparous (127 vs 53) and tended to birth smaller babies. Women who planned a CS viewed breech presentation more negatively, were more worried after the diagnosis, more often sought information from non-professionals and placed more trust in them compared to the professionals involved in their care. Women in the planned CS group were more likely to report a positive birth experience regardless of their eventual birth mode. Women in both groups used the internet to source information related to breech presentation and birth.

Homer et al.^9^ conducted a descriptive exploratory study which examined the experiences of women in New South Wales, Australia, planning a VBB. They reported finding the following themes and sub themes: 1) Reacting to a loss of control, loss of choice, symptoms of stress, feeling trapped, grieving, and bargaining; 2) Wanting the information given to be trustworthy, trust versus mistrust of the information, reacting to scare tactics information, the absence of good information, and needing non-emotive information; 3) Fighting the system/seeking support for a vaginal birth, courage and resolve to fight, taking control, non-fearful clinicians, and support to get back on the path to normal birth; 4) The importance of having a go, labor as a rite of passage, labor as a rite of choice, the way women want to experience birth, for herself and for the baby. They concluded that women planning a VBB valued relevant, consistent and clear information. Women also desired the right to choose VBB and be supported in their decision with high quality care.

Morris et al.^4^ explored women’s experiences of breech birth in Western Australia. The authors identified five distinct stages that women experienced, often multiple times and in no particular order, when diagnosed with a breech presentation, these were: reacting, information, bargaining, decision making and acceptance. Utilizing Foucauldian concepts of power and knowledge, interview transcripts were examined to identify power dynamics. Clinicians were perceived to use knowledge as a way of enforcing disciplinary power and by women as a means of arming themselves to fight to regain power and autonomy. In this study midwives were viewed as navigators of a system perceived as medicalized and restrictive. The study highlighted that disciplinary power was used by clinicians heavily in the information stage of the breech experience, and during labor and birth. Clinical practice improvement recommendations included information provision/sharing between clinicians and women, and better access to VBB experienced and supportive clinicians.

Petrovska et al.^10^ reported the quantitative findings of a multinational online survey which explored the experiences of women planning a VBB. A total of 204 women from Australia, New Zealand, the United Kingdom, the United States, Germany and South Africa participated. An online survey was circulated for nine months on closed breech social media groups. The study found that 80.8% of participants were happy with their birth choices and 89.4% would attempt a VBB if a subsequent pregnancy presented breech. A total of 41.8% reported being referred to a VBB skilled clinician after their diagnosis while 42.3% reported seeking out such a clinician themselves. Only 56.7% reported feeling supported by their care provider and 42.3% felt supported by family and friends.

Petrovska et al.^11^ focused on how women sourced VBB supportive clinicians, the quality of information and level of support they received, and reported the qualitative findings of the study (Table 1). Responses were thematically analyzed, coded and categorized and included, among others, the following themes: Seeking the chance to try for a VBB; Encountering coercion and fear; Putting the birth before the baby; Dealing with emotional wounds; Searching for information and support; and Traveling across boundaries and overcoming obstacles in the system. Based on their findings the authors concluded that inadequate systemic and clinical support hinders access to options of care and balanced information for women pursuing a VBB.

Petrovska et al.^12^ explored how social dialogues regarding risk influence women’s choice for a VBB. Thematic analysis was conducted on data generated by interviews undertaken with 22 women in 2013 in New South Wales, Australia. Eight main themes were derived: Confidence in the birthing body and challenges to this belief; Society’s medicalized view of birth; The ‘horror’ of birth; Dealing with imputed rationality; Dealing with criticism of their competence to make decision; Trying to convince the unconvinced; Seeking information for better understanding; and Seeking support from new social networks.

A grounded theory study by Thompson et al.^13^ explored birth mode decision making for term breech. Parents were asked to recount their experiences. Two couples and four individual parents participated. Two main themes were derived: A framework of possible influences on decision making including partner relationships, family and friends, health professionals, personality and personal birth culture, shared experiences, and time available for decision-making. The second theme was mortality salience and was found to be dominant in every parent’s narrative.

The final article by Toivonen et al.^14^ was a case control study based in Finland comparing women who had experienced a breech birth with those who had experienced a cephalic birth. Women were matched by medical and obstetric history and age, as close as possible. The study found women who experienced a breech birth tended to report a more positive birth experience. However, they appeared to have less choice regarding their birthing position and analgesia. They were more than twice as likely to experience an episiotomy (63.5% vs 30.4%) and undergo oxytocic augmentation (83.5% vs 47.9%) than women who experienced a cephalic birth.

### Synthesis findings

Eighty-five findings and interpretive statements were extracted from the eight articles for inclusion in this review. From these, 16 subcategories emerged and were then grouped into four major synthesized categories. Together, the findings illustrate the challenges women with a breech presentation face and highlight a gap between the information and care women with a breech presentation want and what is being provided in mainstream maternity care.

### Major synthesized category one: Women who desire a vaginal birth may experience a range of negative emotions such as feelings of disempowerment, loss, uncertainty and a sense of isolation

Two subcategories and eight findings form this category which describes the emotions women have reported throughout their breech experience. Consistently, studies reported women planning a VBB felt stripped of choices, experienced a sense of loss regarding the rite of passage birth offers or their planned place of birth (i.e. birth center) and feelings of uncertainty. Some women also reported people questioning their competence to make care decisions or experiencing condescension for trying to inform themselves^12^:

*‘I was spoken to by people in a patronizing tone whenever I tried to inform or educate myself. Several times friends said “you've been on the internet haven't you?”, you know, as if to say “aren't you cute!” and “you still don't know what you are talking about!”’*

Some women were told the eventual birth mode was not their choice but was rather up to the attending clinician present at the time of labor and birth^14^:

*‘(Attending hospital) made it clear that it would entirely depend on staff at the time if I rock up in labor and he was still in breech and I knew from reading that one of the biggest drivers for a successful delivery in breech was the experience of the person you've got… and so that whole uncertainty of care at (attending hospital) was really difficult to deal with.’*

Other participants discussed the struggle of dealing with the unknowns of birth and how their limited options affected them emotionally^13^:

*‘You ask yourself what if, God forbid, something happened during birth, afterwards how would you feel, could you forgive yourself?*’ (Participant 5, postnatal father, planned VBB)

*‘I think I was kind of numb to it. Initially ... and I just felt really sad ... and I started to cry. I didn't know that the rite of passage was important to me until the option was taken away. That's what it felt like. The option was taken away.’* (Participant 17; VB)^9^

Table 2 outlines the subcategories for synthesized category one.

**Table 2:** Major synthesized categories, subcategories and findings

*Category*	*Dimension*	*Subcategory*	*Findings*
Major synthesized category 1		Sense of loss and uncertainty	Grieving for lost opportunities
			Dealing with uncertainty
			Dealing with ‘what if’
			Stripped of choice
			Grieving the loss of the ‘rite of passage’
		Feelings of isolation and disempowerment	Feeling a loss of power
			Feeling disempowered
			Feeling isolation
Major synthesized category 2		VBB viewed as a selfish act	Accusations of selfishness
			Feelings of selfishness
		Bullying and dictatorial behavior	Bullying and scare tactics
			Lack of choice
			Clinician stonewalling
			Being dictated to
			‘Not your decision’
		Coping with negative perceptions	Ignoring the negativity of others and trusting your body
			Dealing with the condescension of others
			Mostly negative view of VBB
		Dealing with fears and anxieties	Emotional fallout
			Coping with your own fears
			Became worried when breech diagnosed
		Horror stories	Negative stories
			Taking on others’ experience
		CS is best	VBB presented as dangerous
			Horror stories reinforcing negativity
			Taking the risk from the baby
			VBB will threaten the baby’s life
			Having to deal with the CS is best mentality
			CS seen as ‘safest’
			VBB presented as an unviable option
Major synthesized category 3	Decision-making process and influences	Fact finding and filtering	Seeking information after disparagement
			Seeking information
			Collating the findings
			Available information negatively geared
			Women found detailed statistics they were provided with useful for decision making
			Information sourced on the internet was seen as helpful for birth mode decision-making
			Felt information sourced online was from reliable sources
			Satisfied with the information at the outpatient clinic
			Relied on clinician and family/friends
			Sought internet-based information
			Social media sharing
		Time: for and against	Taking the time to weigh your options
			Time is not necessarily on my side
	Breeching the system	Navigating a restrictive system	Systemic obstacles
			Compromising with clinicians
			Working within the system’s parameters
			Sought out a skilled clinician themselves
			Fear for future breeches
			Referred to a clinician skilled in VBB
			Challenging the patriarchy is difficult
		Supportive factors	Supported by care provider
			Supported by family and friends
			Would have liked to have spoken to women who had been through similar experiences
			Relied on clinician support only
			Felt midwives devoted enough time to them
			Midwives supportive of woman’s choice
			Seeking support elsewhere
			Social media support networks
		Strong sense of self belief	Coping with the withdrawal of support
			Believing against the odds
			Wanting the opportunity to try a VBB
		Finding and appreciating balance	Finding balance
			Thankful for the opportunity to try
			Appreciating balanced information
Major synthesized category 4		Birth choices	Happy with birth choices
			Would attempt a VBB in a subsequent pregnancy
			Positive birth experience
			Mostly positive view on VBB
			Felt their wish of mode of birth was taken into account
			Handled birth well
		Looking back at the birth	Felt strong and happy during labor and birth
			Reported positive memories of childbirth
			Felt final decision on birth mode was their own
			Had a say in birth position
			Choice in being up or lying down in labor
			Choice in pain relief
			Not allowed to follow her wishes for birth position
			Clinicians sometimes push for undesired interventions

### Major synthesized category two: Women who experience a breech presentation at term experience significant pressures to conform to expectations of medical professionals and their families due to perceptions of risk related to breech birth

This category was developed from six subcategories and 22 findings. It reveals the external pressures, mentalities and behaviors women experience. Women reported feeling selfish for wanting a VBB or faced accusations of selfishness if they planned one and were often told ‘horror’ stories. One woman stated^12^:

*‘I was really looking forward to that whole experience of childbirth and everything else. And all of my friends are like “you're mad to want to do it naturally”. People said I was being selfish, but I was being selfless.’*

Another^13^:

Interviewer: *‘Did you feel like there was really a choice to make?’*

Participant: *‘Not without being selfish. To me it would have been selfish to go for a VBB because that is what I wanted. I would have felt selfish at putting my baby at risk, in my mind, so to me there wasn't that much of a choice.’* (Participant 12, postnatal mother, planned ELCS)

Some women, even years after their birth, were still coming to terms with the emotional toll and conflict it produced in their relationships^11^:

*‘It was a very difficult experience for my partner and I, who weren't 100% reconciled on the decision I made to try and deliver. The effects of this continued after the birth, too. It's taken two years and another baby (head down, born naturally) to heal some of those emotional wounds.’*

Several women reported being told they had no choice, or that they or their fetus would die if they did not comply with the directions or recommendations of their care provider^11^:

I was not happy with the threats and bullying which continued into labor - in the complete absence of any medical problems whatsoever I should add, it was a textbook breech/vertex twin birth. [They said] *“You have to get on the bed for a VE (vaginal examination) - you don't have a choice, your babies are going to die, you are going to die, why did you come here if you don't want us to help you, your kids will be left without a mother…”* ,

Also^12^:

*‘Well, it (VBB) was presented but was presented as you could die ... you'll die or your baby might die and I was like ok that's probably not something I want to do then. And even with me talking about it, it was like: “Well why would you want to have that option when I've just told you your baby might suffer, why would you want to talk about it?”. So, it did kind of make me want to discuss it as an option because obviously I was risking my child's life but it was just c-section really (CS5).’*

For full details of the subcategories see Table 2. The full compilations of illustrations for the findings are available (by study) in the Supplementary file.

### Major synthesized category three: Breech birth decision making in a limiting system

Major category three consists of six subcategories and 34 findings. This category was divided into two dimensions: Decision making process and influences, and Breeching the system. Birth mode decision making is known to comprise a complex interplay between several internal and external factors^4,13^. The majority of women in these studies sought information from multiple sources, particularly internet-based information. Women reported feeling that while information sourced on-line was useful in aiding decision-making, it was not always from reliable sources. However, women reported information received from midwives was helpful and influenced their decision-making more frequently than from other sources (doctor or family and friends)^10^:

*‘I read a lot, the Primary midwife helped me –I went to the library at (Tertiary hospital) and got out that breech women wise by Maggie Banks, which I would recommend to anyone who has a breech baby. I love that book, I watched “A breech in the system” (film), I joined Breech birth Australia and New Zealand, I joined coalition for breech birth and I just started to read a lot and I, for myself made the decision that to me the benefits of a CS do not outweigh the risks. That was just I did not want major abdominal surgery just because my baby is malpositioned.’* (VBB1)^4^

In one study, 85.8% of women expressed a desire to hear from other women who had previously experienced a breech presentation^10^ and some, as mentioned in the above quote, actively joined social media groups dedicated to breech presentation, seeking information and support from women with previous experience of a breech presentation or birth.

Women continued to express a preference for vaginal birth, many holding a deep-seated belief in their ability to birth their ‘breechling’ vaginally. However, navigating the maternity care system can be problematic. Women were able to identify barriers to their desire for a vaginal birth such as unsupportive clinicians, lack of birth mode options presented, and negative information or birth stories^4,9^. One woman expressed her concerns for mothers of future breech presenting fetuses due to a lack of skilled and willing clinicians^11^:

*‘I feel it's a shame there is not more education and support for new doctors coming through. They can't support us mums of breechlings if they aren't supported themselves. I'm genuinely fearful that the option of VBBs will die out as the skills are being lost as CS has become the norm.’*

In order to circumvent these obstacles, women independently sought information from multiple sources and supportive practitioners, sometimes travelling 8 hours for care, if they were not referred on by their original carer^4,10^.

Factors women reported to be used in aiding their decision making included detailed statistics and the information and support provided by clinicians, particularly^4,10,11,14^.

One woman describes the discussion she had with her husband after an appointment with a consultant obstetrician (identified by her midwife) who presented her with the risks of VBB4:

*‘He (her husband) said “how many attempts on a VBB end up in a CS?”. And Dr K said “It's roughly 60%”. We were sitting in the car and he said to me “Well 60% is high” and I said “Yeah but we're the 40 ... that's how I have to look at it ... I'm not the 60” … to me the benefits of a CS do not outweigh the risks.’* (VBB1)

This demonstrates this woman’s strong sense of self-belief. She trusted her body’s ability to give birth the way that aligned with her views and preferences.

For many women, midwives played a significant and positive role in their experiences. They were viewed as figures of support, influenced decision-making and aided system navigation when planning a VBB^4,10,14^. Women appreciated finding balance in the information provided by midwives and the care options they were presented^4^:

*‘My midwife was awesome, she said to me “You don't have to have a caesarean you know”. And knowing she was on my side was everything ...’*

Self-sought information was also viewed as an important factor in navigating limits within the healthcare system. In order to understand the boundaries she had to operate in, one woman explained^4^:

*‘You can find a lot of their (tertiary hospital) protocols on Google™ of what their doctors have to follow. And I stumbled across the breech birth one and I found that really informative, so that I knew if I was going to do this, what the parameters were.’* (VBB8)

While for some women time to weigh their options was important^4,13^, one woman stated^13^:

*‘Part of me thinks that perhaps if I had known, all along, that she was breech ... then I probably would have prepared myself or maybe done a bit more research to be more inclined towards a vaginal birth. But on the other side I think perhaps actually I would have had much more time to get used to the idea that it was not the best route.’* (Participant 12, postnatal mother, planned ELCS)

Women expressed appreciation for clinicians who provided them with support, balanced information and the opportunity to attempt the birth mode they desired, even if eventual birth mode differed (i.e. CS instead of a VBB)^4^:

*‘It didn't work as a vaginal birth which was disappointing as my first birth was natural and lovely, but I'm ok with it because I tried everything to turn it and deliver it. A c section was my last option but that is ok. Baby is here now and I have no regrets because at least I tried. I would have felt completely cheated if my only option had been a caesar and I would have felt like I had failed.’*

The subcategories can be viewed in Table 2 with the corresponding illustrations available in the Supplementary file.

### Major synthesized category four: Overall satisfaction with the decision to plan a vaginal breech birth

The final major category comprises two subcategories and 14 findings outlining women’s reflections on their birth and birth-related choices. Full details of the subcategories can be viewed in Table 2. In Toivonen et al.^14^, in their comparison of breech to cephalic births, women in the breech birth group more often had positive reflections of their birth, but less likely to feel they had a say in the position they assumed in labor and birth (37.5% vs 42.3%). This finding was echoed by another study with women reporting not being allowed to assume their desired birth position and clinicians insisting on certain, unwanted interventions^4^:

*‘He (doctor) was really pushing for me to have an epidural and me to have a caesar ... he got really frustrated that I wasn't listening to him I think because when he examined me internally, I felt like her was being so rough ... so much more painful than the actual labor.’* (VBB7)

This demonstrates that women with a breech presentation are expected to conform to the preferences of their care provider. They also experienced an episiotomy at a significantly higher rate than women with a cephalic fetus (63.5% vs 30.4%)^14^. However, a substantial portion of women remembered their birth as positive, felt strong and happy during labor and many women^10^ stated they would attempt a vaginal birth in subsequent pregnancies if the fetus was in a breech position.

Birth reflection was also common. Women who sought a vaginal birth, despite describing instances in which they weren’t given a choice (i.e. birth position or birth location), women were mostly happy with their birth mode decision-making and viewed their births positively^9^:

*‘I felt really proud of my birthing experience. I feel proud that nobody put me off from trying. I think even if it did end up a C-section, I would have been ok with that. Because if it happened [intrapartum CS], it was obviously required. But we had the chance. The fact that she came out in the end is just a bonus. I suspect that it really helped me bond with her [baby]. I was able to pick her up straight away and hold her close to me. It was a very positive experience.’* (Participant 17;VB)

## DISCUSSION

This review provides a synthesis of existing literature regarding women’s experiences of breech presentation and birth mode decision-making. This synthesis highlights the emotional, social and systemic pressures experienced by women related to breech birth mode decision-making.

### Autonomy and breech birth

There is consistent reporting across the literature of biased, counselling, coercive and bullying behavior that highlights the medicalized, risk-focused and paternalistic culture in modern maternity care^4,15,16^. Woman-centered care and respect of bodily autonomy has been a focus of healthcare education for decades^15,17^. Clinicians report respecting women’s autonomy^16^, however this is not always reflected in practice. Jenkinson et al.^16^ explored women’s, midwives’ and obstetricians’ experiences of women declining recommended care. They identified three inter-related themes of valuing the woman’s journey, the clinician’s line in the sand and escalating intrusion^16^. Clinicians espouse respect for women’s right to self-determination (i.e. autonomy), however they also acknowledge that there was a figurative ‘line in the sand’ that women’s choices can cross (i.e. in declining recommended treatment)^16^. Women ‘crossing the line’ reportedly elicited feelings of conflict in clinicians as they perceived the woman’s decision as having the potential to adversely impact on the fetus. This resulted in clinicians employing gradually more intrusive behaviors to change women’s minds – encompassed in the subthemes of Manipulation, Punishment and Judgement, Badgering and Assault^16^. These themes, which show some similarities to the findings of this review, highlight a wider systemic issue of the continued mistreatment of women, particularly during childbirth. This raises concerns related to valid consent and, depending on one’s viewpoint, a violation of human rights. While this is an extremely important issue that needs to be addressed – full discussion of this problem is outside the scope of this review, as such discussion will focus on women with breech presenting fetuses.

There is an expectation for women to follow the recommendations of their obstetric providers. For women with a breech presentation at term, this is often a CS birth or birthing in lithotomy. If women resist, they may be perceived to be valuing ‘natural birth ideologies’ over the safety of their fetus^18^ and experience the intrusive behaviors described by Jenkinson et al.^16^. However, despite these stressors and pressures, women desiring a VBB have described being happy or proud of their birth. Hannah et al.^19^ conducted a follow-up study three months after the TBT exploring postnatal outcomes and maternal satisfaction with their childbirth experience. They found that women in the planned CS group indicated that, while they felt reassured about the health of their infant according to planned birth mode, they were more likely to report disliking their birth mode. Women in the planned VBB group more often liked being an active participant in their birth^19^.

A study by Cook and Loomis^20^ concluded that women’s recollections of birth, be they positive or negative, were associated strongly with feelings of choice and control rather than the minutiae of their birth experiences. These findings, along with those of this review, highlight the importance of a woman-centered approach to breech care and the importance of inclusive decision-making.

### The breech dichotomy and clinicians’ experiences (or lack thereof)

While research^21,22^ indicates CS reduces short-term neonatal morbidity and mortality, long-term outcomes are similar regardless of birth mode^23^. Research describing clinician attitudes and experiences of breech presentation and management indicate that while many view breech presentation along a spectrum of normality, the majority of participants reported a lack of experience in facilitating VBB due to a lack of opportunity and exposure^24,25^. So while evidence and clinical guidelines support the practice of VBB, many clinicians who would like to provide safe support for woman desiring a VBB, lack the skill to do so due to changes in practice which were cemented by the TBT recommendations^3,26^.

As the safety of VBB is directly related to the skill and experience of the birth attendant, the lack of experience reported by clinicians is problematic for planned and undiagnosed breech births and likely contributes to the feelings of conflict and uncertainty in clinicians^16^. Breech birth skills are mainly taught in emergency study days alongside complications such as shoulder dystocia. Midwives in Sloman et al.^25^ felt this was an inappropriate approach to teaching breech skills and could lead to panic in clinicians and likely interrupts physiological birth processes.

Clinical practice guidelines support the skill development of both midwives and obstetricians in VBB^27-29^. A Delphi study exploring breech presentation reported that participants endorsed breech birth being taught as a ‘normal’ skill instead of an emergency event^30^. Participants also suggested incorporating upright breech techniques, breech-specific progress measures and optimal mechanisms as well as the establishment of breech teams to support the wider team in maternity care settings^30^. Upright breech birth techniques have been shown to reduce the rate of neonatal injuries, the incidence of birth manoeuvres and the rate of serious perineal damage^31^. This may be due to a move away from the traditional medical practice of lithotomy positioning for breech birth^32^. Lithotomy positioning has been shown to significantly increase the risk of severe perineal trauma, even for women with cephalic presenting fetuses^33^. But with the rarity of VBB in the majority of settings and breech birth continuing to be taught as an emergency, how are clinicians meant to develop skills and increase their confidence?

Maternity facilities that do not routinely offer VBB have been urged by many academics to provide their staff with VBB learning opportunities as inevitably, VBB will continue to occur in the clinical setting^34-47^. Simulation based training, has been suggested^34,48^ as a way of addressing the lack of confidence and skills among clinicians who attend births – midwives and obstetricians alike. Simulation based training is now a typical contemporary midwifery and obstetric training tool^48^. It provides practitioners the opportunity to practice, and make mistakes in a supported environment, with no clinical outcome ramifications^48^. Nevertheless, there is no evidence suggesting that simulation-based training can replace the significance of lived experiences obtained in the clinical environment^49^.

### Midwives and breech

Morris et al.^4^ describe how women understood that the diagnosis of a breech presentation would change their pregnancy and birth experience. The findings of this review depict diagnosis of breech presentation as a stressful event in the current maternity and societal climate due to persistent negative attitudes, despite the small actual risk of planning a VBB^27^. Clinicians are vital in aiding women in navigating changes to their pregnancy and birth experiences^50^, for example upon the diagnosis of a breech presentation. Women identified midwives as a positive influence on their experience, through their ability to provide information, support and referral to obstetric clinicians willing to provide a balanced approach to birth mode counselling^4,10,14^.

Research related to existing breech services or teams, indicates that midwives are involved in the counselling and care of women with a breech presentation^51-53^. Midwives were at times responsible for initial discussions with women around breech presentation and basic assessments. They were also involved in birth counselling^52,53^, mostly in regard to labor and birth positions and available pain-relief options^51^. Australian research reports, that in a service which employs a multidisciplinary breech team, midwives and obstetricians are equally responsible for facilitating breech birth^54^.

### Improving breech care

Research exploring clinicians’ experiences of providing care to women planning a VBB in a service where VBB was offered regularly provides insight into what breech birth counselling has the potential to be when clinicians have the experience and confidence to support women in their desire for a ‘non-standard’ birth^54^. Participants highlighted the importance of exploring the woman’s knowledge and feelings of breech presentation and tailoring the discussion of safety and risk to the woman’s individual circumstances based on their medical and obstetric history. Participants felt it was important to address the TBT as this was one of the most widely referenced and accessible texts but also because the findings may cause concern amongst women^54^. Continuity of care was also highlighted as important as was maintaining a calm demeanor and not sensationalizing the situation so as not to alarm women^54^. For women’s experiences to improve, a culture shift is needed along with a more woman-centered approach to maternity care. This is important because birth experiences impact, both negatively and positively, on women’s well-being, mental health and by extension on their family unit^57^. Strategies seen to facilitate VBB were education and training of clinicians to increase confidence and clinical skills; facilitate a calm, supportive and collaborative approach to VBB and careful counselling and selection of women^47^. These recommendations are consistent through the literature and can be achieved through the development of breech teams or dedicated breech services^34-46,^ ^53,56^.

Specialized or dedicated breech services offer non-biased birth mode counselling, ECV and support for women’s birth mode choices under a multidisciplinary team^3,4^. They have been shown to improve the uptake and success of ECV and increase the number of women choosing the option of a VBB, therefore reducing the CS rate for breech presentation when certain criteria are met^51,53^. A reduction in the rate of CS for breech has the potential to reduce the incidence of Severe Acute Maternal Morbidity (SAMM)^1^. Midwives and obstetricians work collaboratively to provide women with balanced care options and are currently in operation in Australia and throughout the United Kingdom^3^ and have been suggested as a potential solution to the power inequalities between medical professionals and women and the existent breech birth skill deficit^4^. This model of care has the potential to provide women with actual birth mode choices and increase women’s satisfaction of their care and experience.

Such services, if set up well, would also allow for junior practitioners to learn and eventually teach much needed breech birth skills in a safe, supportive environment and provide lived experiences lacking in other forms of training^3^. If facilities decline to opt for this approach to breech birth, then the onus is on individual clinicians to obtain the skills necessary to safely facilitate a VBB in both traditional (lithotomy) and upright or lateral positions. This would not only provide practitioners with an invaluable skills set, it would facilitate support of women’s autonomy and provide flexibility for women to assume a birth position which may be more acceptable to them. This can be achieved through breech birth courses such as those offered by Breech Without Boarders (www.breechwithoutboarder.org), Breech Birth Network (www.breechbirth.org.uk) and the Becoming a Breech Expert (BABE)^57^ courses. Obtaining these skills would, at least for some professionals, erase the ‘line in the sand’ described by Jenkinson et al.^16^, allowing them to support and respect women’s autonomy.

### Limitations

While a systemic approach was conducted for this review, there is the possibility of applicable studies being missed. For example, due to the language parameter, perspectives of non-English speaking women are unlikely to be adequately represented despite the inclusion of some multinational studies.

## CONCLUSIONS

This review examined women’s experiences of breech birth-mode decision-making. Women with a breech presenting fetus at term experience a complex range of emotions and internal and external pressures due to an ingrained perception of risk around birth, particularly breech birth. Midwives were seen as helpful throughout the breech experience. Speciality breech services may provide the opportunity for clinician upskilling, support and respect of women’s autonomy through the uptake and improved success of ECV and appropriately selected women in achieving a VBB. This in turn would reduce the incidence of Severe Acute Maternal Morbidity.

## Data Availability

The data supporting this research can be found in the supplementary content.
